# Gastroenteritis and respiratory infection outbreaks in French nursing homes from 2007 to 2018: Morbidity and all-cause lethality according to the individual characteristics of residents

**DOI:** 10.1371/journal.pone.0222321

**Published:** 2019-09-24

**Authors:** Philippe Gaspard, Anne Mosnier, Loic Simon, Olivia Ali-Brandmeyer, Christian Rabaud, Sabrina Larocca, Béatrice Heck, Serge Aho-Glélé, Pierre Pothier, Katia Ambert-Balay

**Affiliations:** 1 Hospital Hygiene Service, Rouffach Hospital Center, Rouffach, France; 2 UMR 6249 Chrono-Environnement, University of Franche-Comté, Besançon, France; 3 Open Rome, Paris, France; 4 Coordination Centre for Nosocomial Infection Control, Eastern Regions, Nancy University Hospital, Nancy, France; 5 Department of Epidemiology and Infection Control, Dijon University Hospital, Dijon, France; 6 University Burgundy Franche-Comté, AgroSup Dijon, PAM UMR A 02.102, Dijon, France; 7 National Reference Center for Gastroenteritis Viruses, Laboratory of Biology and Pathology, University Hospital, Dijon, France; INSERM, FRANCE

## Abstract

**Background:**

Gastroenteritis (GE) and respiratory tract infection (RTI) outbreaks are a significant issue in nursing homes. This study aimed to describe GE and RTI outbreaks with infection and all-cause lethality rates according to the individual characteristics of nursing home residents.

**Methods:**

Clinical and virological surveillance were conducted (2007 to 2018). Virus stratifications for the analysis were: outbreaks with positive norovirus or influenza identifications (respectively NoV+ or Flu+), episodes with no NoV or influenza identification or testing (respectively NoV- or Flu-). Associations between individual variables (sex, age, length of stay (LOS), autonomy status) and infection and lethality rates were tested with univariate and Mantel-Haenszel (MH) methods.

**Results:**

61 GE outbreaks and 76 RTI oubreaks (total 137 outbreaks) were recorded involving respectively 4309 and 5862 residents. In univariate analysis, higher infection rates and age were associated in NoV+, NoV-, and Flu+ contexts, and lower infection rates were associated with longer stays (NoV+ and NoV-). In MH stratified analysis (virus, sex (female/male)) adjusted for LOS (<4 or ≥4 years), the odds of being infected remained significant among older residents (≥86 years): NoV+/male (Odds ratio (OR_MH_): 1.64, 95% confidence interval (CI): 1.16–2.30) and Flu+/female and male (respectively OR_MH_: 1.50, CI: 1.27–1.79 and 1.73, CI: 1.28–2.33). In univariate analysis, lower autonomy status (NoV+, Flu+ and Flu-) and increased age (Flu+) were associated with higher lethality. In MH adjusted analysis, significant OR_age_ adjusted for autonomy was: Flu+/ ≥86 years compared with <86 years, 1.97 (1.19–3.25) and OR_autonomy_ adjusted for age for the more autonomous group (compared with the less autonomous group) was: Flu+, 0.41 (0.24–0.69); Flu-, 0.42 (0.20, 0.90).

**Conclusion:**

The residents of nursing homes are increasingly elderly and dependent. The specific infection and lethality risks according to these two factors indicate that surveillance and infection control measures are essential and of high priority.

## Introduction

Gastroenteritis (GE) and respiratory tract infection (RTI) outbreaks represent a significant burden of illness in nursing homes. Viruses cause the majority of these outbreaks, and noroviruses and influenza viruses are the most common pathogens [1,2].

Previous studies have suggested that viral respiratory infections and norovirus outbreaks are a common cause of hospitalization or death, particularly among elderly individuals [3–5].

The impact of outbreaks has been described in terms of both frequency and epidemiology, but little is known about infection rates and all-cause lethality in GE and RTI nursing home outbreaks in relation to the individual characteristics of the residents [6]. The residents of these institutions are increasingly elderly and dependent, and the impact of this trend on the seasonal outbreak burden requires in-depth investigation. The results of studies focused on this issue could yield valuable information for nursing homes, allowing them to adapt their infection control strategies, in particular for improved assessment of infection risk.

Our objective was to describe GE and RTI infection and all-cause lethality rates according to the individual characteristics of nursing home residents (sex, age, length of stay, autonomy status), and to identify specific susceptibility patterns related to these types of viral outbreaks in these facilities.

## Materials and methods

### Design and setting

The present study explored outbreaks in 14 sites (28 units with geriatric nursing home activities for a total of 1121 beds) caring for dependent people in southern Alsace (an area in north-eastern France). Data were collected between September 2007 and August 2018 [7,8].

Each site was geographically independent and autonomous for social and care management. Units were located within the larger sites and were defined as a place having a dedicated team at one location.

### Outbreak inclusion and individual characteristics

During outbreaks at one site, only the residents in the units with confirmed cases were included. Outbreak inclusion depended on institutional alert to the hygiene team. Surveillance was done in each unit independently, and the members of staff had to inform a physician or charge nurse when two or more potential related cases of pneumonia or GE were observed within four days and when three or more cases were observed for other RTI. Units also had to inform the hygiene team when these threshold values were exceeded. For influenza, the first suspected case led to a local alert and the hygiene team was contacted. A practitioner from the hygiene team collected the information and evaluated the clinical signs, the virology information and the epidemiological context with the physician in the affected unit. The detected cluster was only put under surveillance if the hygiene team considered that there was a potential outbreak phenomenon. The duration of 4 days was in relation with the national protocol with alert to the authorities when 5 cases occurred within 4 days [9,10]. On a local level and in addition to the clusters reported to the authorities, clusters with at least 3 cases within a period of seven days in one unit could be recorded if they were reported to the hygiene team. Because several outbreaks could potentially occur in the same unit during the surveillance period, a resident could be included repeatedly in different clusters. As a result, the observed patterns reflected the characteristics of an institutional population with longitudinal and pluriannual exposures.

### Nursing home resident data

Personal information and clinical information was collected by a practitioner from the hygiene team directly from the residents’ health care records. Personal information was collected for all those present the first day of the outbreak. The collected information included: month and year of birth, sex, date of arrival at the nursing home and autonomy status. The autonomy status of residents in French nursing homes is assessed using the AGGIR scale (Autonomy Gerontology Groups Iso-Resources), which is the legal instrument for evaluating dependency in the elderly and whose primary purpose is the allocation of means and resources [11].

With the AGGIR scale, autonomy is classified into 6 Iso-Resource Groups (GIR): GIR 1 (bedridden or armchair-bound persons, mental functions seriously altered and requiring continuous presence), GIR 2 (bedridden or armchair-bound persons, mental functions not totally altered and requiring assistance in most activities of daily living, or mental functions altered with preserved ability to get around), GIR 3 (preserved mental autonomy with partially preserved motor autonomy and assistance several times a day for physical autonomy), GIR 4 (moves around the home and sometimes assistance for washing, dressing, physical activities or eating), GIR 5 (only occasional assistance for washing, meal preparation, and housework), GIR 6 (autonomy for essential tasks of daily living). In outbreaks where the influenza virus was identified, influenza vaccination status and oseltamivir prescriptions were recorded as well. A file is transmitted in the Supporting Information with all previous data (S1 Data).

### Case inclusion and lethality study

GE was defined as the sudden onset of vomiting and/or diarrhea over a 24 h period: (i) diarrhea ≥3 episodes, (ii) and/or vomiting ≥3 episodes, (iii) or diarrhea or vomiting <3 episodes with two or more other symptoms (diarrhea, vomiting, stomach ache, abdominal cramps, nausea, fever, mucus in stools) [1].

RTI presentation in older adults may be atypical, like for other acute illnesses in this age group [12]. We used the recommended definitions for RTI surveillance in geriatric units, divided in 3 subcategories: (i) common cold syndromes or pharyngitis (at least two of the following criteria: runny nose or sneezing, stuffy nose (i.e. congestion), sore throat or hoarseness or difficulty swallowing, dry cough, swollen or tender glands in the neck (cervical lymphadenopathy)), (ii) influenza-like illness (both the following criteria must be met: fever AND at least three other symptoms (chills, new headache or eye pain, myalgia or body aches, malaise or loss of appetite, sore throat, new or increased dry cough)) and (iii) lower respiratory tract infection (both of the following criteria must be met: at least two respiratory signs or symptoms (new or increased cough, new/increased sputum production, O_2_ saturation <94% or reduced >3% from baseline, abnormal lung examination (new or changed), pleuritic chest pain, respiratory rate ≥25 breaths/min AND one or more constitutional signs/symptoms (fever, leukocytosis, confusion, acute functional decline)) [13–16]. Infection corresponding to one of these three subcategories was included in this study and classified as RTI.

For both infection types, the practitioner from the hygiene team obtained clinical information from the patient’s health care records, and members of the health care team were consulted if necessary to complete any missing information. At the end of the episode (within seven days after the last identified case), case inclusion as exposed and not infected (ENI) or exposed and infected (EI) was determined with a resident physician.

In order to study the lethality, the presence of each infected resident was evaluated once at least 56 days after the last case of each outbreak. Each resident was followed up retrospectively during eighth 7-day interval (I_n, n = 1 to 8,_ total 56 days, between the date of onset of symptoms and the fifty-sixth day of the studied period) with three different possibilities: present (alive and officially residing in the institution), lost to follow-up (alive at the date of departure but no longer residing in the institution (return home, transfer to another institution)) or death (death recorded in the health care record). The dates of death and lost to follow-up were recorded.

### Virological investigation

Testing for the virus was not systematic and was decided by the physicians in each institution in the presence of clinical signs.

For GE, stool samples were sent to the National Reference Centre for Gastroenteritis Viruses in Dijon for laboratory testing, as previously described [8]. For RTI surveillance, rapid tests were used to identify the influenza virus. The rapid immunoassay diagnosis tests used for influenza detection were: Clearview® Exact Influenza A and B (Inverness Medical, Cologne, Germany) from 2007 to 2014 and InfluenzaTop® (Alldiag, Strasbourg, France) from 2014 to 2018. Given the low sensitivity of influenza rapid tests, they were no longer used once the control measures had been implemented and the influenza outbreak was under control. Samples were also occasionally sent to hospital laboratories or to the National Reference Centre for Influenza Viruses to detect viruses with real-time RT-PCR [17]. Most testing targeted the norovirus (NoV) and influenza virus, but other tests were occasionally performed by the National Reference Centre for Influenza Viruses (rhinovirus, respiratory syncytial virus, human metapneumovirus, parainfluenza 1, 2, 3 and 4, and coronavirus) and the National Reference Centre for Enteric Viruses (rotavirus, astrovirus, and adenovirus).

Because testing for the viruses was variable (from one institution/physician to another, not used in some outbreaks, types of virus sought) and considering the poor sensitivity of the rapid influenza tests, these two sources of data were used to define the epidemiological context of confirmed outbreaks. Consequently, individual cases were included consistently in all episodes according to clinical signs and medical evaluation. When virus testing was negative, the clinical signs were recorded and medical evaluation was used as previously to classify the included residents as infected or not infected.

The epidemiological context of each outbreak was defined according to whether the virus had been identified or not. One or more positive samples led to the qualification of a NoV (NoV+) or flu (Flu+) context. The other episodes were qualified as flu or NoV outbreaks with no specific identification or testing (NoV- and Flu-).

Flu and NoV contexts did not eliminate other potential enteric or respiratory pathogens.

### Data analysis

Sex, age, length of stay (LOS, in years) and autonomy status were described for all exposed residents. Influenza vaccination and oseltamivir administration rates were calculated for confirmed influenza outbreaks. Dichotomous or categorical variables were expressed as percentages. In univariate analysis, the categories were specific in order to obtain a precise description of the age and LOS variables. Class intervals were 5 years for age and one year for LOS. The residents classified as GIR 4 to 6 (sometimes, occasional and no assistance) were grouped together because they were few.

For the multi-level analysis with 2x2 tables, a median value was used to define the two-level age categories. For LOS, assessing the longest stays was necessary to identify the effect of longer exposure in a nursing home. Consequently, a four-year cutoff was chosen to create the two categories. For autonomy, the two most dependent categories (GIR ≤ 2) were grouped together and compared with the more autonomous categories (GIR ≥ 3).

The outbreak epidemiological contexts were used with the four categories: NoV+, Flu+, NoV- and flu-. Other GE or RTI viruses were occasionally identified, but the number of results was too limited to develop separate analyses. However, all the results are available in the tables about the virus investigations along with NoV and influenza identifications.

For the different categories, infection rate (EI/Exposed Residents (ER), in percentage) was calculated according to sex, age group, LOS and autonomy status. To investigate all-cause lethality and define the appropriate period for the 56-day monitoring (D_1 to 56_), the all-cause lethality rate per 7-day interval (LR_n_/I_n, n = 1 to 8_) was calculated: (number of deaths during interval I_n_/(EI alive the first day of n^th^ studied interval minus lost to follow-up EI during the interval I_n_)*100).

Seeing as successive clusters could occur within the same site, potentially influencing all-cause lethality, the serial interval in days (SI_d_) was calculated. The SI_d_ was the time period between the onset of symptoms of the last case in initial outbreak (N) and the onset of symptoms of the first case in the following outbreak (N+1). Investigations were performed when SI_d_ was shorter or equal to the length of the previous D_1 to 56_ and the following parameters were evaluated for these specific situations: number of episodes, residents infected in both outbreaks, and death among the identified individuals.

Finally, according to the death rate and the impact of successive outbreaks, the number of 7-day intervals (N.I_n_) to take into account was defined, and the all-cause lethality rate was analyzed during these periods (I_1 to_ N^th^.I_n_ or D_1 to 7*N_).

All-cause lethality rates were calculated with the following formula: (number of deaths from D_1 to 7*N_/(EI number at D_1_ minus lost to follow-up among EI during the period D_1 to 7*N_)*100). Estimation of the turnover rate per 7-day interval among the infected residents was calculated on the base of the LOS (median in years) with the following formula: (proportion of discharged residents: 50.0% in the case of the median)/[(median LOS *365)/7)]. The average rate of residents discharged per 7-day period was calculated: [(number of lost to follow up during the period D_1 to 7*N_/number of exposed and infected residents at D_1_)/N 7-day interval]*100.

As some residents were included in several outbreaks during the surveillance, the observations were not completely independent; non-parametric tests were used as a result. In univariate analysis, Chi-square or Fisher exact tests (expected number of frequencies fewer than 5) were used to compare infection and lethality rates according to the studied parameters and the odds ratio was calculated by median-unbiased estimation. The Kruskal-Wallis test was used to compare median values. Confidence intervals for medians were calculated with bootstrap methods.

Covariate adjusted analyses were performed with two tables (2x2). The respective impact of each individual factor was tested with Mantel-Haenszel chi-squared tests. The equality of the stratum odds ratios was tested with the Woolf test of homogeneity. Finally, for each virus context, multiple tables (2x2) were generated and tested with confounding variables, effect modifiers or covariables. Statistical analyses were done using R for Mas OS X version R 3.4.1 software with RStudio version 1.0.153. A file is transmitted in the Supporting Information with all R codes and the packages used (S1 R Codes). Differences were considered significant at p ≤ 0.05.

## Ethical aspects and consent

The French Data Protection Authority approved data collection and analysis (DE-2013-074) and the local ethics committee (Espace Local de Réflexion Ethique, Centre Hospitalier de Rouffach) approved the study protocol (ERLE-32). According to the French law for biomedical research and human experimentation, individual written consent was not required from the patients or their relatives for data collection. Each year, the referring local practitioner of the study coordinated with the doctors working in the nursing home. At the beginning of the surveillance period, information regarding participation in the study was displayed in the family vising area, including a document about their right to access and rectify personal data. After collection, data were rendered anonymous. No specific authorization was needed to retrospectively analyze anonymous data collected during routine care in the context of routine surveillance.

## Results

A total of 137 outbreaks were recorded in the 14 sites. RTI outbreaks were more frequent than GE outbreaks (76 outbreaks and 5862 exposed residents vs. 61 outbreaks and 4309 exposed residents, respectively). Overall, 7643 of the exposed residents were women and 2528 were men. The median age was 86.7 years old (interquartile range: 81.1–91.0 years).

Virus investigations (respectively 389 samples for RTI and 143 for GE with all the detailed results in S1–S4 Tables) confirmed a considerable number of norovirus-related GE outbreaks (34/61) and influenza-related RTI outbreaks (46/76). For GE outbreaks, 2524 residents were in a NoV+ context versus 1785 in a NoV- context, and for RTI outbreaks, 3479 residents were in a Flu+ context versus 2383 in a Flu- context.

For GE surveillance in the NoV+ context, there were 1093 EI residents versus 1431 ENI residents, whereas in the NoV- context, there were 583 EI residents versus 1202 ENI residents. Therefore, the infection rate was higher in the NoV+ context (43.3%) than in the NoV- context (32.7%, (odds ratio (OR): 0.63, 95% confidence interval (CI): 0.56–0.72), *p* < 0.001).

For RTI surveillance, the rates of infection were similar with and without confirmed influenza: 31.5% (N = 1095 EI residents /3479 exposed residents) vs. 30.5% (N = 728 EI residents/2383 exposed residents, OR: 0.96, CI: 0.85–1.07, *p* = 0.47). Moreover, infection rate in the NoV+ context was higher than the three other contexts: NoV- (OR: 0.63, CI: 0.56–0.72), Flu+ (OR: 0.60, CI:0.54–0.67) and Flu- (OR: 0.58, CI: 0.51–0.65).

In univariate analysis, certain individual characteristics were associated with significant variations in the infection rate (S5 Table). The infection rate increased with age (except in the Flu- context) and, decreased with LOS during GE outbreaks. The covariate adjusted analysis revealed specific significant effect modification according to sex (NoV+) and LOS (NoV-) (S6 Table). In analyses stratified according to virus and sex, age adjusted for LOS remained significant for Flu+ and NoV+ outbreaks (males). In NoV- context, the effect modification of LOS remained significant (Table 1). Finally, when autonomy was included and adjusted for age (virus, sex, LOS stratification), the less autonomous residents (female/LOS<4 years/age<86/GIR 1–2) were affected more severely by Flu+ outbreaks with specific effect modification according to age (S7 Table).

**Table 1:** Infection rates with 4 level analysis (virus, sex, length stay, age) in outbreaks according to individual characteristics.

	Factor 1	Factor 2	Variable	Infection rates	Odds Ratio	*p* ^a^	Infection rates	Odds Ratio	*p*
	Female	Length of stay (years)	Age (years)						
Virus context					**NoV+**^c^			**Flu+** ^d^	
		<4	<86	43.8 (537)		0.48	27.6 (700)		**<0.001**
			≥86	45.9 (732)	1.09 (0.87–1.36)		35.5 (1076)	1.45 (1.18–1.78)	
		≥4	<86	37.7 (257)		0.06	25.4 (331)		**0.002**
			≥86	45.6 (386)	1.38 (1.00–1.91)		35.8 (495)	1.63 (1.20–2.23)	
Odds Ratio		-	-	-	1.17 (0.98–1.41)	0.09	-	1.50 (1.27–1.79)	**<0.001**
Homogeneity test		-	-	-	-	0.24	-	-	0.51
MH ^b^ adj. Odds Ratio		-	-	-	1.18 (0.98,-.42)	0.09	-	1.50 (1.27–1.79)	**<0.001**
	Male		Age						
Virus context					**NoV+**			**Flu+**	
		<4	<86	40.3 (278)		**0.03**	25.9 (355)		**0.001**
			≥86	51.6 (161)	1.57 (1.06–2.33)		36.4 (231)	1.63 (1.14–2.34)	
		≥4	<86	27.6 (127)		0.12	24.3 (206)		**0.02**
			≥86	41.3 (46)	1.84 (0.90–3.74)		38.8 (85)	1.98 (1.15–3.40)	
Odds Ratio		-	-	-	1.70 (1.21–2.39)	**0.002**	-	1.73 (1.29–2.33)	**0.004**
Homogeneity test		-	-	-	-	0.70	-	-	0.56
MH adj. Odds Ratio		-	-	-	1.64 (1.16–2.30)	**0.006**	-	1.73 (1.28–2.33)	**0.004**
	Female		Age						
Virus context					**NoV-**^e^			**Flu-**^f^	
		<4	<86	38.7 (377)		0.35	30.0 (433)		0.55
			≥86	35.5 (527)	0.87 (0.66–1.14)		31.9 (772)	1.09 (0.85–1.41)	
		≥4	<86	23.1 (169)		0.11	27.0 (211)		0.58
			≥86	30.1 (261)	1.47 (0.95–2.31)		29.6 (379)	1.13 (0.78–1.66)	
Odds Ratio		-	-	-	0.99 (0.79–1.26)	1.00	-	1.10 (0.89–1.36)	0.39
Homogeneity test		-	-	-	-	**0.05**	-	-	0.87
MH adj. Odds Ratio		-	-	-	-	-	-	1.10 (0.89–1.36)	0.39
	Male		Age						
Virus context					**NoV-**			**Flu-**	
		<4	<86	31.5 (219)		0.91	31.8 (264)		0.98
			≥86	30.2 (106)	0.94 (0.56–1.55)		31.2 (173)	0.97 (0.64–1.47)	
		≥4	<86	18.1 (94)		**0.02**	26.6 (113)		0.20
			≥86	40.6 (32)	3.07 (1.25–7.49)		39.5 (38)	1.80 (0.82–3.91)	
Odds Ratio		-	-	-	1.28 (0.82–1.97)	0.32	-	1.1 (0.78–1.61)	0.60
Homogeneity test		-	-	-	-	**0.02**	-	-	0.16
MH adj. Odds Ratio		-	-				-	1.11 (0.77–1.60)	0.64

^a^ *P* values *(*Pearson's Chi-squared or Fisher's Exact Test or Mantel-Haenszel X-squared)

^b^ Mantel Haenszel

^c^ Norovirus context

^d^ Influenza context

^e^ No Available Norovirus Identification or Research

^f^ No Available Flu Identification or Research.

The study of lethality rates in infected residents over the 56 days after onset indicated that there were significant variations for RTI but no change for GE (Table 2). Significant differences appeared after 28 days in the context of Flu+ outbreaks and other RTI outbreaks.

**Table 2:** Lethality rates during the 8 seven-day intervals after the inclusion of infected residents.

	Gastroenteritis surveillance			Respiratory Tract infection surveillance		
Seven-day intervals	Lethality rates % (infected residents, lost to follow up)	Odds Ratio	*p* ^a^	Lethality rates % (Infected residents, lost to follow up)	Odds Ratio	*p*
	NoV+ ^b^			Flu+		
1	0.5 (1093, 0)	-	0.42	2.5 (1094, 1)	-	< 0.001
2	0.3 (1088, 0)	0.61 (0.12–2.61)		2.2 (1067, 0)	0.87 (0.49–1.53)	
3	0.6 (1085, 0)	1.20 (0.35–4.32)		2.1(1042, 2)	0.85 (0.48–1.51)	
4	0.3 (1079, 0)	0.62 (0.12–2.63)		1.8 (1019, 1)	0.71 (0.38–1.30)	
5	0.3 (1076, 0)	0.62 (0.12–2.64)		0.9 (1000, 1)	0.36 (0.16–0.75)	
6	0.9 (1072, 1)	2.02 (0.70–6.65)		0.5 (990, 1)	0.21 (0.07–0.50)	
7	0.4 (1062, 0)	0.83 (0.20–3.26)		0.5 (985, 0)	0.21 (0.07–0.50)	
8	0.4 (1058, 0)	0.83 (0.20–3.27)		0.8 (980, 0)	0.32 (0.14–0.70)	
	NoV- ^c^			Flu- ^d^		
1	1.2 (583, 0)	-	0.85	2.3 (728, 0)	-	< 0.001
2	0.7 (575, 1)	0.58 (0.14–2.00)		1.4 (711, 0)	0.60 (0.26–1.31)	
3	0.9 (570, 1)	0.73 (0.21–2.37)		1.0 (701, 0)	0.43 (0.16–1.01)	
4	0.7 (565, 0)	0.59 (0.15–2.03)		1.0 (694, 0)	0.43 (0.16–1.02)	
5	0.4 (560, 1)	0.31 (0.04–1.33)		0.9 (687, 0)	0.38 (0.13–0.92)	
6	0.9 (558, 0)	0.75 (0.21–2.42)		0.3 (681, 0)	0.13 (0.02–0.47)	
7	0.5 (553, 0)	0.46 (0.09–1.71)		0.4 (679, 0)	0.19 (0.04–0.59)	
8	0.7 (550, 0)	0.61 (0.15–2.09)		0.2 (676, 0)	0.07 (0.03–0.34)	

^a^ *P* values *(*Pearson's Chi-squared or Fisher's Exact Test)

^b^ Norovirus context

^c^ No Available Norovirus Identification or Research

^d^ No Available Identification or Research

The analysis of successive or simultaneous clusters in the same institutions was performed when the time period between the onset of symptoms of the last case in outbreak N and the onset of symptoms of the first case in outbreak N+1 was ≤56 days (S8 Table). 44 of the 137 outbreaks (32.12%) were identified, and 194 of the 3499 exposed and infected residents contracted multiple infections. The percentage of exposed and infected residents implicated in more than one virus stratification was 11.09% ((194*2)/3499). Moreover, two deceased residents were included in the NoV-Na and Flu lethality analyses because death occurred within 56 days for both infections. The analysis of virus stratification of the 44 outbreaks showed the absence of successive clusters for the same category. The same analysis for the first four 7-day intervals (Days 1 to 28) showed the respective values: 26 outbreaks (18.98%), 117 residents ((6.69% ((117*2)/3499)), one dead resident.

Finally, according to the higher lethality impact during the first four 7-day intervals and to limit the impact of successive clusters in the same site, all cause lethality rates were studied according to individual parameters for the four 7 days intervals with the respective number of included deaths: NoV+: 17, NoV-NA: 20, Flu+: 90 and Flu-NA: 41.

According to the virus context, the difference in median LOS was not significantly different depending on the virus context: NoV+ (2.4, 95% CI: 2.3–2.6), NoV- (2.1, 95% CI: 2.0–2.3), Flu+ (2.3, 95% CI: 2.2–2.5), Flu- (2.2, 95% CI: 2.1–2.4), *p* = 0.08). The estimated weekly turnover rate for all infected residents (length stay median = 2.3 years, 95% CI: 2.2–2.4) was 0.41% per 7-day period, totalling 1.64% for four 7-day intervals. The average weekly rate of discharged residents (lost to follow-up) was equal to: [10/3499/8]*100 = 0.035% or 0.14% for the four 7-day intervals.

Univariate analysis showed significant differences in lethality according to virus surveillance. Lethality in the NoV+ context was lower than the three other levels: Nov- (odds ratio (OR): 2.25, (95% confidence interval (CI): 1.17–4.40), Flu+ (OR: 5.65, CI: 3.43–9.89) and Flu- (OR: 3.75, CI: 2.15–6.85).

According to the surveillance type (GE or RTI), the lethality rates differed significantly: 1.6% versus 3.4% (respectively NoV+ and NoV- contexts, OR: 2.24, CI: 1.16–4.39, *p* = 0.02) and 8.3% versus 5.6% (respectively Flu+ and Flu-, OR: 0.67, CI: 0.45–0.97, *p* = 0.04).

In univariate analysis (S9 Table), low autonomy status in the NoV+, Flu+ and Flu- contexts was most significantly associated with increased all-cause lethality, and age was associated with higher lethality in the Flu+ context. In the adjusted analysis, no significant statistical differences were identified in GE outbreaks. For RTI episodes, the adjusted analysis showed that autonomy had a significant impact when adjusted for sex, age or LOS (Flu+ and Flu-NA) and that age had a significant impact when adjusted for sex, autonomy or LOS (Flu+) (S10 Table).

In Table 3, the specific effects of age or autonomy were tested. Significant OR_age_ adjusted for autonomy were: Flu+/age ≥86 years (compared with the <86 group), 1.97 (1.19–3.25). OR_autonomy_ adjusted for age were for GIR 3–6 (compared with GIR 1–2): Flu+, 0.41 (0.24–0.69); Flu-, 0.42 (0.20, 0.90).

**Table 3:** Lethality rates with 3-level stratification (virus, age, autonomy) in outbreaks according to individual characteristics.

	Factor	Variable	Lethality rates	Odds Ratio	*P*^a^	Lethality rates	Odds Ratio	*p*
	Age (years)	Autonomy						
Virus context				**NoV+** ^c^			**Flu+** ^d^	
	<86	1–2	1.2 (259)	-	0.63	6.3 (268)		0.31
		3 to 6	0.5 (218)	0.43 (0.01–3.72)		3.4 (145)	0.54 (0.17–1.41)	
	≥86	1–2	2.4 (375)	-	0.22	12.6 (412)		**0.002**
		3 to 6	0.9 (233)	0.37 (0.05–1.50)		5.1 (257)	0.37 (0.19–0.68)	
Odds Ratio	-	-	-	0.36 (0.08–1.16)	0.15	-	0.42 (0.24–0.70)	**0.001**
Homogeneity test	-	-	-	-	0.87	-	-	0.50
MH ^b^ adj. Odds Ratio	-	-	-	0.36 (0.10–1.30)	0.12	-	0.41 (0.24–0.69)	**<0.001**
	Autonomy ^e^	Age						
Virus context				**NoV+**			**Flu+**	
	1–2	<86	1.2 (259)	-	0.38	6.3 (268)	-	
		≥86	2.4 (375)	2.03 (0.59–9.68)		12.6 (412)	2.12 (1.22–3.86)	**0.01**
	3 to 6	<86	0.5 (218)	-	1.00	3.4 (145)	-	
		≥86	0.9 (233)	1.76 (0.14–55.66)		5.1 (257)	1.46 (0.53–4.74)	0.61
Odds Ratio	-	-	-	2.12 (0.71–7.95)	0.27	-	1.90 (1.17–3.20)	**0.01**
Homogeneity test	-	-	-	-	0.87	-	-	0.50
MH adj. Odds Ratio	-	-	-	2.05 (0.65–6.50)	0.29	-	1.97 (1.19–3.25)	**0.008**
	Age	Autonomy						
Virus context				**NoV-**^f^			**Flu-**^g^	
	<86	1–2	4.2 (165)			5.9 (187)	-	0.14
		3 to 6	1.0 (103)	0.25 (0.01–1.47)	0.16	1.8 (113)	0.31 (0.04–1.19)	
	≥86	1–2	4.3 (207)			8.2 (257)	-	0.16
		3 to 6	2.9 (104)	0.68 (0.14–2.37)	0.76	4.2 (167)	0.50 (0.19–1.16)	
Odds Ratio	-	-	-	0.45 (0.12–1.26)	0.21	-	0.43 (0.19–0.89)	**0.04**
Homogeneity test	-	-	-	-	0.44	-	-	0.63
MH adj. Odds Ratio	-	-	-	0.45 (0.15–1.35)	0.16	-	0.42 (0.20, 0.90)	**0.02**
	Autonomy	Age						
Virus context				**NoV-**			**Flu-**	
	1–2	<86	4.2 (165)	-	1.00	5.9 (187)	-	0.46
		≥86	4.3 (207)	1.02 (0.37–2.97)		8.2 (257)	1.41 (0.67–3.13)	
	3 to 6	<86	1.0 (103)	-	0.62	1.8 (113)	-	0.32
		≥86	2.9 (104)	2.77 (0.32–80.49)		4.2 (167)	2.30 (0.53–17.29)	
Odds Ratio	-	-	-	1.30 (0.52–3.40)	0.73	-	1.55 (0.80–3.16)	0.25
Homogeneity test	-	-	-	-	0.44	-	-	0.63
MH adj. Odds Ratio	-	-	-	1.26 (0.51–3.12)	0.65	-	1.59 (0.81–3.13)	0.19

^a^ *P* values *(*Pearson's Chi-squared or Fisher's Exact Test or Mantel-Haenszel X-squared)

^b^ Mantel Haenszel

^c^ Norovirus context

^d^ Influenza context

^e^AGGIR scale: autonomy Gerontology Group Iso-Ressources

^f^ Not Available Norovirus Identification or Research

^g^Not Available Flu Identification or Research.

Finally, despite the low number of residents and deaths per category, and consequently the limited robustness of the results, autonomy adjusted for age with stratification according to virus, sex and LOS showed that the effects were higher among subgroups of less autonomous residents (female or male/LOS<4 years/GIR 1–2) in Flu+ outbreaks, and there was also higher mortality in the small subgroup of autonomous men with LOS ≥ 4 years (higher mortality) (S11 Table).

In the Flu+ context, data regarding vaccination status and oseltamivir prescriptions were available but not used in this study.

## Discussion

In the present study, surveillance data obtained during GE and RTI outbreaks in nursing homes were used to construct stratified analyses and to identify specific infection and all-cause lethality rates according to the residents’ individual characteristics.

The infection rates observed here were similar to those found in previous studies of NoV and Influenza outbreaks (odds of being infected during a Flu+ outbreak were around 40% less than during a NoV+ outbreak). Reported infection rates were close to 30.0% in influenza outbreaks and 40.0% in NoV outbreaks [18–20].

Older age appeared to increase the likelihood of GE and influenza infection, with increasing rates among older residents. Age is a well-known factor for influenza and norovirus severity in the elderly and in nursing homes [21,22]. For NoV, the highest incidence estimates (5-year age strata) was found in the ≥85 year-category (approximately 800 men and for 1,400 women per 100,000 inhabitants). In our study, univariate analysis (NoV+) showed that the odds of being infected were 1.5 to 1.6 times higher if a resident was older than 85. Moreover, an adjusted analysis of GE outbreaks highlighted different effects among subgroups of residents according to sex and LOS. Indeed, multiple and/or repeated exposure to GE viruses while institutionalized may lead to susceptibility or possible increased immunity in some residents [23]. For the sex variable, two factors could explain the effect: a possible selection bias with men reporting mild infections less than women (particularly in the <86 years subgroup) or that male susceptibility was different (age, LOS, immunity,…). A German study from 2013 also reported a greater impact in women [21]. Moreover, when age analysis was stratified by sex and LOS, no significant impact was observed in women; the only significant differences were fewer infections in men in the <86-subgroup (except in the NoV- with LOS <4 years).

For the RTI outbreaks, sex and LOS variables did not have a significant effect. In residents older than 86, the odds of being infected in Flu+ context were 1.5 times higher for women and 1.7 for men. In univariate analysis, contrary to the other virus contexts where odds ratios were rarely above 2, residents over 95 years old had increased odds of infection of ≥ 2.8 compared with the 70-year-old category, and for the 100 year-old group the odds were approximately 3.8. In the Flu+ context, autonomy adjusted for age (virus, sex and LOS stratification) revealed a possible increase in infection rates among less autonomous residents. A previous study found that when elderly residents were exposed to the A(H3N2) virus, there were higher rates of infection and reinfection, and more significant effects on the institution than with other influenza types/subtypes. In the community, the relative illness ratio (RIR) in the ≥75 year-group reflected this different impact: 0.09/2009-2010 season (AH1N1 predominant) and 0.59/1999-2000, 0.48/2004-2005, 0.42/2011-2012 (AH3N2 predominant) [22,24].

The incidence of influenza infection and the associated risks were well described by age group, but the specific impact according to age was not studied. The results of this work highlighted the specific age distribution of influenza illnesses among the nursing home residents and the more significant impact among the older residents. This specific susceptibility could be a critical factor in the institutional exposure and dissemination of influenza and could partly explain the high infection impact in the elderly institutional population.

In this work, autonomy status was not the main factor associated with infection (no significant impact in GE and in Flu- contexts). However, in Flu+ outbreaks, a high level of dependency was associated with a higher risk of falling ill. This observation implies that staff could play a role in the spread of infection (highly dependent and less mobile residents are less likely to contaminate themselves) or that the more active residents may be less fragile and/or have a greater involvement in the recommended infection control measures. Finally, improving compliance with personal hygiene measures both for nursing staff and residents might be expected to have a beneficial effect on infection rates. Previous studies identified higher NoV infection rates in highly dependent individuals, but the results were not adjusted for age and LOS to take into account the potential correlation with the autonomy status [20].

Lethality is difficult to assess in nursing homes because death is frequent. Our GE and RTI episodes occurred during the winter seasons, and there are possible interactions between outbreaks and increased mortality at this time of the year [25]. The all-cause lethality rate of the infected residents in our study reflected global mortality including GE and RTI outbreaks and the global epidemiological context. Not surprisingly, a higher all-cause lethality rate was observed in the influenza contexts, as reported in previous studies [25–27]. Age and autonomy had similar effects in the different contexts, but in GE and to a lesser degree in Flu- outbreaks, the relatively small number of deaths could have limited the power of the statistical tests. In nursing homes, residents are generally discharged due to death. The number of residents lost to follow up was low (0.14% in the first 28 days), so the 7-day interval turnover rate calculated on the base of the median LOS provides a good indication of the average case fatality rate. The lethality rate for NoV+ outbreaks was similar to the estimated 7-day interval turnover rate (1.6%) indicating that this context had a limited impact on the death rate.

The all-cause lethality rate was most affected by age and autonomy. Both individual characteristics were significant in the Flu+ outbreaks, and autonomy adjusted for age was significant in the Flu- episodes. The influence of age on mortality in a context of influenza has already been described: a very high mortality rate (831/100,000 inhabitants) was reported in persons 90 years of age and older compared with those aged 65–69 years (23/100,000 inhabitants) [28]. In our univariate analysis, the higher risk was observed in the ≥90 group whose risk of death was at least 2.6 higher than the <70 group. When adjusted for autonomy, the impact of age was not significant in more autonomous residents in the Flu+ context and not at all in the Flu- context. The opposite analysis (autonomy adjusted for age) showed higher global impact in the less autonomous group (Flu-) or only in the ≥86 age group (Flu+). Age and autonomy are a reflection of resident’s level of frailty. Clinical frailty scores were not used in this study, but in a previous study of patients with critical illness, they were associated with greater mortality, regardless of age [29]. This suggests that in addition to age, autonomy can be a valuable indicator for the assessment of outbreak impact in outbreak surveillance. Other studies have suggested that age and certain comorbidities are independent risk factors for the influenza mortality rate or that mortality increase according to the number of risk factors [28, 30]. Comorbidities and underlying diseases of various severities could reflect overall frailty and consequently the risk of death. In nursing homes, information about autonomy and age are easier to collect and interpret than data on comorbidities. These various approaches should be evaluated and compared in the goal of optimizing risk assessment among nursing home residents.

The present work has two main limitations. First, the virus information was incomplete (limited identification, mainly influenza rapid tests for the RTI). Consequently, some episodes in the levels with no available identification may also have been associated with influenza or norovirus, and multiple contaminations could have been underestimated or not taken into account. Moreover, vaccination and oseltamivir prescriptions were recorded but not included because the influenza genotype was not determined and identification was limited.

Secondly, the deaths of uninfected residents were not recorded in this protocol even though such data would have provided valuable information about the global epidemiological context.

In conclusion, specific susceptibility patterns were observed among exposed residents. In this cohort of nursing homes, infection rates varied according to virus, sex, length of stay and age, and there were major differences in lethality depending on virus, age and autonomy score. The collected data were easy to record and could be used to improve the characterization of seasonal outbreaks in nursing homes, whose residents are particularly vulnerable. Finally, as the average age and dependency level of residents continues to increase, subsequently increasing the risk of infection and death, health care staff will have to be increasingly vigilant during seasonal outbreaks and targeted interventions should be implemented.

## Supporting information

S1 Data (CSV)

S1 R Codes (R)

S1 Table (XLSX)

S2 Table (XLSX)

S3 Table (XLSX)

S4 Table (XLSX)

S5 Table (XLSX)

S6 Table (XLSX)

S7 Table (XLSX)

S8 Table (XLSX)

S9 Table (XLSX)

S10 Table (XLSX)

S11 Table (XLSX)
